# Metallothionein-3 as a multifunctional player in the control of cellular processes and diseases

**DOI:** 10.1186/s13041-020-00654-w

**Published:** 2020-08-25

**Authors:** Jae-Young Koh, Sook-Jeong Lee

**Affiliations:** 1 Neural Injury Research Center, Asan Institute for Life Sciences, University of Ulsan, College of Medicine, Seoul, 05505 Republic of Korea; 2 Department of Neurology, Asan Medical Center, University of Ulsan, College of Medicine, Seoul, 05505 Republic of Korea; 3 Department of Bioactive Material Science, Jeonbuk National University, Jeonju, Jeollabuk-do 54896 Republic of Korea

**Keywords:** Autophagy, Alzheimer’s disease, Lysosome, Metallothionein-3, Neurodegenerative disease, Oxidative stress, Zinc

## Abstract

Transition metals, such as iron, copper, and zinc, play a very important role in life as the regulators of various physiochemical reactions in cells. Abnormal distribution and concentration of these metals in the body are closely associated with various diseases including ischemic seizure, Alzheimer’s disease, diabetes, and cancer. Iron and copper are known to be mainly involved in in vivo redox reaction. Zinc controls a variety of intracellular metabolism via binding to lots of proteins in cells and altering their structure and function. Metallothionein-3 (MT3) is a representative zinc binding protein predominant in the brain. Although the role of MT3 in other organs still needs to be elucidated, many reports have suggested critical roles for the protein in the control of a variety of cellular homeostasis. Here, we review various biological functions of MT3, focusing on different cellular molecules and diseases involving MT3 in the body.

## Introduction

Intracellular transition metals exist in various forms- free or attached to proteins or organelles. Most metals in the cells are involved in diverse cellular function, including the recycling of organelles and proteins as well as the vesicular transport. Abnormal concentration of metals can lead to cytotoxicity by redox imbalance. Many researchers have reported that iron, copper, manganese, and zinc participate in the Fenton reaction and contribute to the regulation of reactive oxygen species (ROS) production. In the pathophysiology of dementia, these metals are entangled with various proteins and deposited on specific brain lesions. Proper concentration of intracellular transition metals is pivotal to maintain the normal biological activity in cells.

Metallothioneins (MTs) are proteins that bind or release cellular transition metals, an interaction that depends on specific cell situations. MTs were first reported by Vallee and Margoshe as Cd-binding protein from horse renal cortex [1]. Initial studies focused on the structure of the protein but soon extended to biochemical and functional features and gene regulation strategies. MTs have four subfamilies in human, designated as MT1, MT2, MT3, and MT4. Each subtype is differentially distributed in tissues. Since their discovery in horse kidney, Binz and Kägi classified members of 15 MT families in different species, ranging from yeast to vertebrate and plants. Individual species show a whole array of sequences of different length, number and position of cysteine, and intercalating residues in the protein [2].

MTs have a high affinity for zinc and copper, and their structure can change depending on the number of bound metals, which contributes to functional differences. MT1 and MT2 are found ubiquitously in a broad range of organs; however, MT3 and MT4 are specifically expressed in the central nervous system and stratified squamous epithelia, respectively [3]. Recently, MT3 was reported as having certain specific functions in tissues other than the brain [4]. Therefore, this review addresses the intracellular role of MT3 and its relationship with disease, specifically focusing on its relationship with intracellular zinc in various cells.

### Structural characteristics of MT3

In general, protein structure reflects its functional characteristics. Therefore, structural modification of the protein may lead to a change in its activity. MT3 is a small protein of molecular weight ~ 7 kDa with up to 20 cysteine residues, a high cysteine amount of almost 30% of their amino acid content [5]. The cysteine residues confer a high chance of binding with essential or transition metals. MT3 has several structural features that distinguish it from other MTs, suggesting that MT3 may differs in function from the other MTs.

Majority of studies have focused mainly on MT1 and MT2. Specifically, most functional studies on these proteins described the regulation of essential metal homeostasis and heavy metal ions detoxification [6]. However, since the discovery of a new role of MT3 as a growth inhibitory factor (GIF) in the brain, specifically in neurons of patients with Alzheimer’s disease [7], the structure and function of MT3 have started gaining a great attention. In the following sections, we focus on describing the structural characteristics of MT3.

#### Common structural characteristics of MT3 with other MTs

**Fig. 1:**
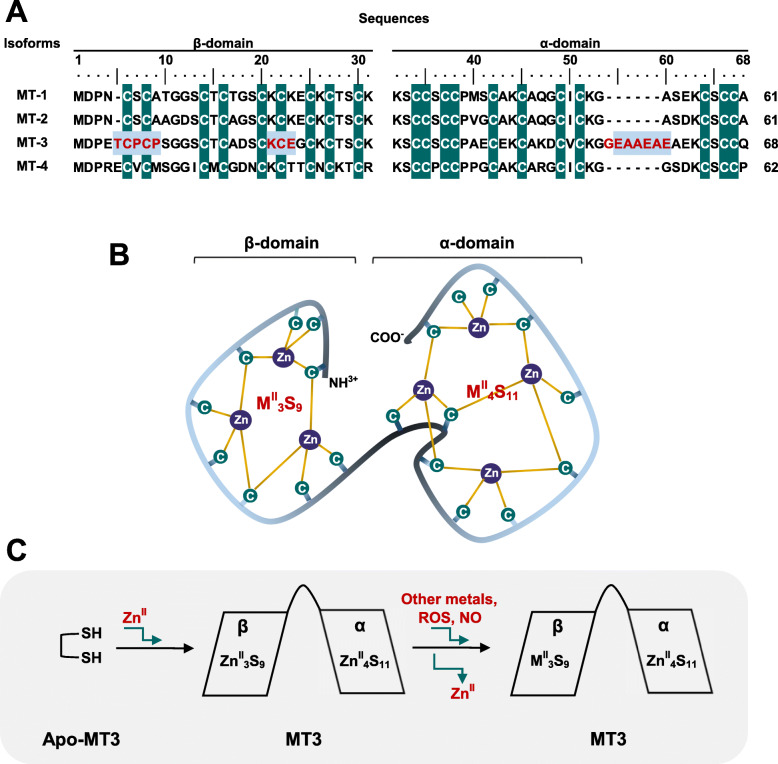
Schematic diagram of MT3 amino acid sequences and structure and its response to oxidative stress. **a** Amino acid sequences alignments of human MT1, MT2, MT3, and MT4. Conserved cysteine residues are highlighted in dark green. The distinctive sequence differences of MT3 from other MTs include an insertion of Thr5, a conservative CPCP (6–9) sequences, and insertion of the charged hexapeptide EAAEAE (55–60). **b** Simplified structure of MT3. The β-domain of the N-terminal contains nine cysteine residues, but α-domain of the C-terminal contains 11 cysteine residues, providing three and four zinc binding sites, respectively. **c** Metal swap in MT3 under oxidative injury. MT3 without any metals (apo-MT3) can bind up to seven zinc. Under ROS or NO stress, three Zn in β-domain can be released by swapping with radicals and the metal exchange ability may protect cells from the oxidative injury

MTs possess heterogeneity among species; however, they have common features in their structures as shown in Fig. 1. The common structure among MTs includes a preserved 20 cysteine residues and two major domains, which wrap around a metal-thiolate cluster known as the α (C-terminal)- and β (N-terminal)-domain [8]. The C-terminal contains eleven cysteines with a thiol group, whereas there are nine cysteines in the N-terminal [8, 9]. The thiol group in cysteine residues of MTs confers a high metal-binding affinity, thus resulting in tetrahedral structural conformation. Hence, each domain of MTs constitutes differential pairs of metal-thiol association- the C-terminal α-domain contain a four-metal cluster (M^II^_4_S_11_) and the β-domain of the N-terminal comprises a three-metal cluster (M^II^_3_S_9_) (Fig. 1b). In addition, all four isoforms of MTs contain at least five conserved Lys residues at their C-terminals; MT1 and MT2 (MT1/2) have eight conserved Lys. Interestingly, MTs lack of aromatic amino acids residues such as His, Phe, Thy, or Trp residues. As previously indicated, the sulfur ligands in cysteine residues provide room for bonding with various metals. Metallation in MTs is mainly found with Zn^II^ and Cd^II^, but also with Hg^II^, Cu^I^, Cu^II^, Ag^I^, Au^I^, Bi^III^, As^II^I, Co^II^, Fe^II^, Pb^II^, Pt^II^, and Tc^IV^ [10]. Especially, MTs may bind up to seven atoms of Zn or Cd [11].

#### Unique sequences of MT3

As mentioned above, MT3 has several unique features of its own in structure, which is absent in MT1/2. First, all known MT3 sequences contain a conserved ‘Thr5-Cys6-Pro7-Cys8-Pro9 (TCPCP)’ motif in their β-domain of the N-terminal (Fig. 1a). Second, MT3 possesses ‘Glu55-Ala56-Ala57-Glu58-Ala59-Glu60 (EAAEAE)’ hexapeptide insert in its α-domain in the C-terminal (Fig. 1a). Third, it has an acid-basic catalysis motif (Lys21-Cys22-Glu23, KCE) in the β-domain (Fig. 1a). Each sequence insert serves its own function.

MT3 is very dynamic. The TCPCP motif of β-domain in MT3 has been reported as the main site associated with its neuronal growth inhibitory activity. Thus, MT3 is also called as GIF protein [12, 13]. N-terminal residues containing TCPCP sequences are more flexible than other regions of the protein, suggesting that the TCPCP sequence in the N-terminal may contribute to the dynamics of the β-domain of MT3 [14]. Initially, TCPCP motif was reported as essential for the GIF activity of MT3. Indeed, the TCSCA (in MT1) and TCTCT (in MT2) double mutations of the TCPCP motif almost completely eliminated the GIF bioactivity [15]. However, neuronal assays have shown that the TCPCP motif alone is inadequate for GIF bioactivity [15]. Notably, of all MTs, only MT3 also possesses Thr5, a residue now associated with its optimal GIF activity [16]. Consistently, a Thr5 to Ser5 mutation or Thr5 deletion (∆Thr5) showed similar effects. It has thus been suggested that hydrogen bonding by Thr5 strengthens the β-domain structure and regulates the GIF activity of MT3.

The EAAEAE (55–60) site located around the C-terminal is actually far from the metal-thiolate cluster; thus, this position is less restricted making adopting alternative conformation possible [15, 16]. Especially, these sequences contribute to interactions between the interdomain linker region and the α-domain of MT3. Study with deletion mutants of EAAEAE revealed that interactions of two hydrogen bonds between Lys31 and Glu4, and Ser33 and Cys38 were lost in the mutants, influencing the movement of the α-domain away from the conformation of the β-domain. However, the different point mutations at this site did not change the GIF [17].

Lastly, KCE (21–23) sequence at the unique acid-basic catalysis motif in the β-domain plays an important role in the interaction with the thiolate group of MTs that leads to the release of zinc ions [18]. However, this sequence in MT3 does not differentially respond to S-nitrosylation as MT-1/2. Of the residues in the site, only Glu23 is important in the specific conversion of nitric oxide (NO) signals into zinc signals, thus sensitizing NO metabolism and/or zinc homeostasis, which eventually regulate neuronal growth-inhibitory activity [19].

### Functions of MT3

**Fig. 2:**
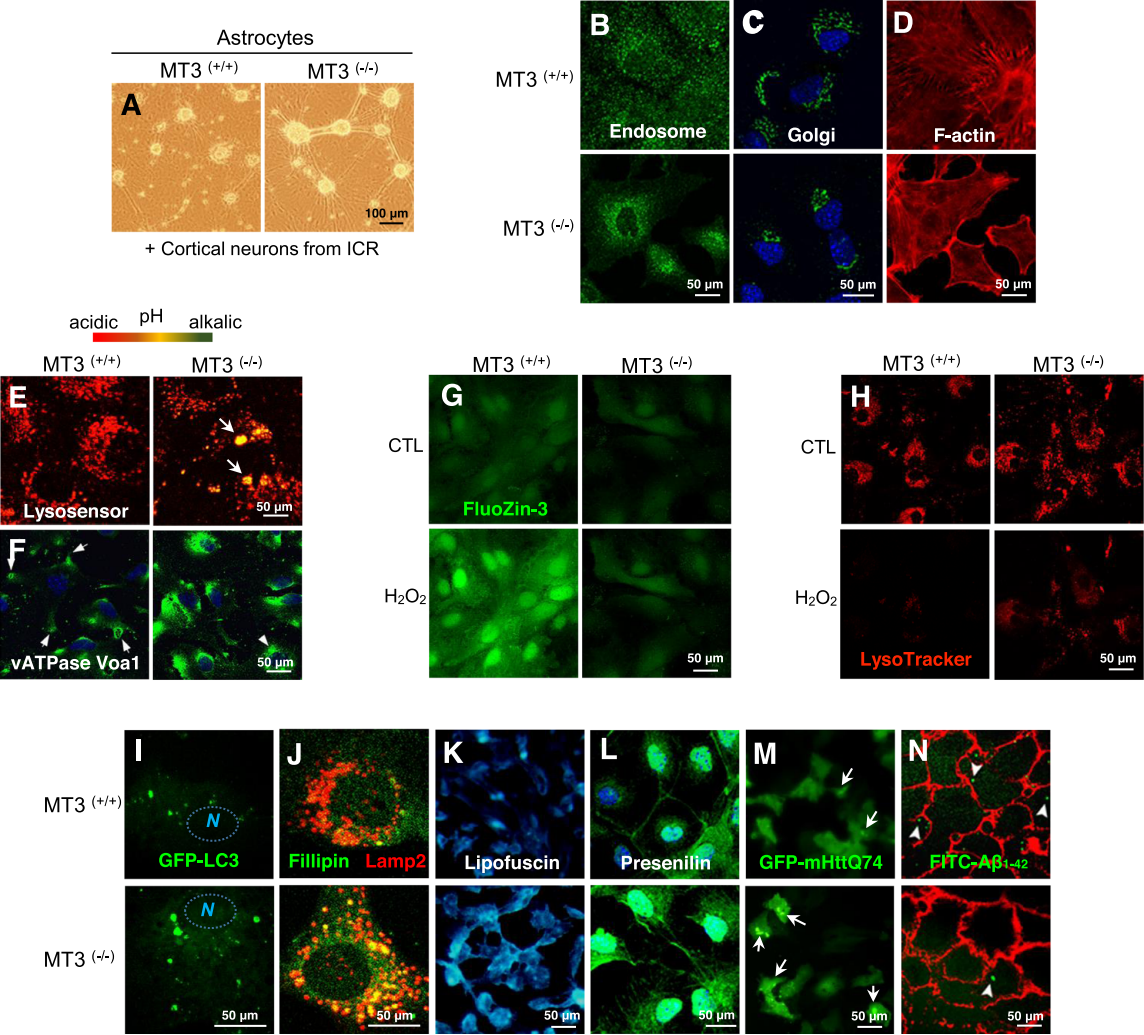
Representative images of various intracellular responses to the absence of MT3. **a** Neuronal outgrowth induced in MT3 knockout (KO) mice. Cortical astrocytes from MT3 wild type (WT, ^+/+^) and KO (^−/−^) mice were used as the feeder cells. Cortical neuronal cells from ICR mice were cultured onto the astrocytes from the indicated sources. **b-d** Confocal images of altered localization and expression of intracellular components, such as endosomes **b**, Golgi **c**, F-actin **d**, in cortical astrocytes from MT3 WT and KO mice. **e** Fluorescence images of lysosensor-stained MT3 WT and KO cortical astrocytes. **f** Confocal images of altered localization and expression of vATPase Voa1 in cortical astrocytes from MT3 WT and KO mice. **g-h** Live-cell confocal microscopic images of WT and MT3-null astrocytes. Cortical astrocytes were exposed to 100 μm of H_2_O_2_ and the intracellular labile zinc was traced using a FluoZin-3 **g**, free zinc staining fluorescence dye, and Lysotracker **h**, a fluorescence dye staining of lysosomes. **i-m** Confocal images of altered localization and expression of intracellular proteins; Cortical astrocytes from WT and MT3-null astrocytes positive for GFP-LC3 **i**, fillipin and Lamp2 **j**, lipofuscin **k**, presenilin **l**, and GFP-mHttQ74 **m**. Cells were stained with filipin (green) **j** and lipofuscin (cyan) **k** against cellular lipids. Lamp2 was used to determine the cellular localization of intracellular fillipin-positive signals. GFP-mHttQ74 (green dot) was overexpressed in cortical astrocytes and its expression was compared between WT and KO of MT3. **n** Live-cell confocal microscopic images of WT and MT3-null astrocytes. The plasma membrane of astrocytes was stained with phalloidin, a membrane stain, and FITC-Aβ_1–42_ was loaded and the intracellular uptake into astrocytes was traced. FITC-Aβ_1–42_ was uptaken into cells 15 min after loading

MT3 maintains the homeostasis of copper and zinc in cells, as other MTs, protecting cells from oxidative stress and regulating cell growth, differentiation, and other normal cellular functions [20]. As shown by our study in Fig. 2a, the finding of MT3 as a neuronal growth inhibitor has attracted the attention of many researchers as MT3 functions slightly different from the other isoforms. Current studies have been expanded beyond investigations of normal MT3 structure and the change that follow altered metal-binding affinity and metal mobilization. MT3 has now been described as complicatedly associated with a wide range of diseases including cancer, diabetes, retinopathy, and various brain diseases. We further describe the major known features of MT3 function, followed by a discussion of associated disease.

#### ROS and MT3

The best-known function of MTs is the control of redox reactions. MTs bind or release various kinds of toxic or redox-active metals that contribute to ROS. Redox control by MTs occurs mainly by scavenging NO, hydrogen peroxide, hydroxyl, and superoxide radicals because MTs contains cysteine sulfur clusters inside the protein, thus easily form deprotonated cysteine, disulfide bonds, and structure changes, which eventually contribute to radical quenching.

MT3 shares many common structures with MT1/2, but there are chemical and structural differences. In particular, MT3 has a more flexible β-domain, making better access to larger molecules to metal-thiolate cluster possible. These might be ascribed to the sequence differences in the N-terminal amino acids of MT1/2 and MT3 that create different changes in cluster structure of the proteins when they react against diverse radicals. To prove this, compared with MT1/2, MT3 was shown to be much more reactive to NO and *S*-nitrosothiols, which are molecules with larger stoichiometric characteristic, than to small molecules such as hydrogen peroxide and superoxide radicals [21]. Similarly, MT3 shows differential responsiveness to ROS, compared with MT1/2. For instance, MT3 was not able to scavenge superoxide derived from hypoxanthine oxidase reaction, but directly captured the hydroxyl radicals from hydrogen peroxide [22]. Moreover, in hyperoxic environment, exogenous and endogenous MT3 reportedly regulate neuronal differentiation and death [22].

There are a great number of sources of ROS in a cell. ROS are consistently generated by diverse normal metabolic processes such as Fenton-type reaction and photolysis of hydrogen peroxide in plant cells. When cells work too hard, the mitochondria get overloaded and ROS builds up beyond the limits. Excess radicals damage DNA and ROS could destruct intracellular organellar membranes and the plasma membrane by lipid peroxidation. MT3 controls not only metals in the cytosol but also metals within many intracellular proteins. The effects of MT3 under oxidative stress conditions remain controversial. In some reports, MT3 showed a strong ROS-scavenging property via swapping intracellular ROS-inducing components with metals in the cysteine-thiol cluster of MT3, thus protecting from damage in stress-conditioned cells [22] (Fig. 1c). However, in other reports, in vivo MT3 knock-out mice and in vitro MT3 knockdown model by siRNA resulted in protective effect against H_2_O_2_-induced oxidative injury [23, 24]. Importantly, MT3 as well as MT1/2, has a high affinity for copper and zinc, which are the major essential metals for normal body metabolism, although most MT1/2 found in vivo exists in the form of Zn_7_-MTs, except when cells are exposed to excess concentration of Cu [25]. However, isolated MT3 is found in a mixed form of Zn and Cu content, usually as Cu(I)_4_-Zn (II)_3 ~ 4_-MT3 [26]. These controversial differences in affinity for metals among MTs family might be due to the β-domain of the proteins. MT3 β-domain is more unstable than α-domain, resulting in better release and exchange with other components in the cytosol during abnormal cellular conditions [7, 27].

There is an interesting report that showed that MT1/2 contribute to increase in ROS-regulated gene expression in oxidative stress conditions, but not MT3 [27]. On the contrary, metal ions did not alter very much MT3 expression, but MT1/2 expression quickly responds to intracellular metal toxicity [27]. This may reflect that the role of MT1/2 is more focused on ROS regulation. However, MT3 has other major biological roles in cells, such as the inhibition of growth [7].

#### Cytoskeleton and MT3

In order for a cell to survive, activation of many signal molecules in the cell is necessary, and sometimes these molecules need to move from one cell to another, or from one specific cellular site to reach their targets. Cytoskeleton proteins play an important role in the heart of all these processes. Besides maintaining the structure and shape of cells [28, 29], cytoskeletal proteins are involved in endocytosis and exocytosis [30]. During signal transport, cytoskeleton cooperates with extracellular connective tissues on the plasma membranes and diverse cytosolic vesicles [28, 30]. The cytoskeleton is also involved in the segregation of chromosomes during cellular division and cytokinesis, regulating the cell cycle [28, 30]. Cytoskeleton is composed of three main components― microfilaments, intermediate filaments, and microtubules. Actin is the major component of microfilaments and can assemble or dissemble for the action in the cell. Tubulin is the component of microtubules [28].

There are reports on nuclear import of MTs by *MT1/2* mRNA associated with the perinuclear cytoskeleton [31]. According to these reports, perinuclear recruits of *MT1/2* mRNA sort cytoskeletal filaments to perinuclear region and are essential for efficient shuttling of the protein into the nucleus during G1 to S phase transition. Many mRNA transcribed in the nucleus are exported to the cytosol. However, *MT-1/2* can be transported to nucleus; however, they do not travel to the cytosol but stay with cytoskeletal-bound polysomes or the cytoskeleton itself [31]. At this position, they import many proteins into the nucleus during G1/S transition to control. Especially, *cis*-acting signals in the 3′ untranslated regions (3′ UTR) of *MT1* has been known to contribute to localization of mRNA into perinuclear region [31]. Specifically, two regions of this protein ―nucleotides 21–36 and 66–76― are the sites of binding with elongation factor 1α (eEF1α) [31]. eEF1α bound to this specific region of *MT1* mRNA anchors the MT1 mRNA to the cytoskeleton in the perinuclear cytoplasm, leading to the synthesis of the protein near to its site of function. Consistent with this, it has been reported that eEF1α binds to the localized actin mRNA [32], and in the case of eEF1γ, with vimentin mRNA [33].

MT3 has also been reported as a possible regulator of cytoskeleton function. El Ghazi and Lahti have separately reported on proteins interacting with MT3 in the mouse brain [34, 35]. Immunoaffinity chromatography over immobilized anti-mouse brain MT3 antibody revealed that heat-shock protein (HSP) 84 (mouse variant of HSP 70), dihydropyrimidinase-like protein 2 (DRP-2), HSP 70, creatine kinase (CK), and β-actin bind to MT3. In particular, MT3 consists of a complex with CK, HSP70, and HSP 84, but not with β-actin or DRP-2. Recently, our group has found a new role of MT3 in the regulation of cAbl activity [36]. In this study, MT3 controls normal actin polymerization (Fig. 2d), and regulates filamentous actin (F-actin) and cAbl binding by contributing to the dissociation of the interactions when cortical astrocytes were stimulated by epidermal growth factor (EGF). Notably, TCPCP motif in the N-terminal of MT3 is the key domain for this effect. Moreover, MT3 also adjusts amyloid β (Aβ_42_) endocytosis in the cortical astrocytes through the regulation of actin polymerization [37] (Fig. 2n). Interestingly, the cytoskeleton is affected in various neurodegenerative disorders such as Alzheimer’s disease (AD), Huntington’s disease (HD), Parkinson’s disease (PD), and amyotrophic lateral sclerosis (ALS) [38]. For instance, altered microtubule assembly and stability in PD [39] and AD [40], excess glutamine production by altered linking vesicles onto the cytoskeleton in HD [41], and damage of motor neurons due to defective cytoskeleton [42] have been demonstrated. In the brain, the cooperative interaction among different types of cytoskeleton proteins is important to move signal molecules through vesicles from the soma to the apical end of a nerve, along the long axons. Therefore, although it has not been fully identified and need to be further studied, MT3-induced control of cytoskeletal filaments may in part contribute to severe neurodegenerative diseases.

#### Cellular organelles and MT3

MTs are present in various locations depending on the cell type. MTs are known to be in cytosols, nuclei, and mitochondria [43]. However, they have been reported to exist in lysosomes [44]. MTs are small in size and less than 10 kDa, so like other small-sized proteins, they can be introduced into the nucleus by passive diffusion through the water-soluble portion of the nuclear pore complex [45]. MTs imported into the nucleus control cells by upregulating transcription factors related to various intracellular functions, particularly the cell cycle, cell growth, cell differentiation, and ROS regulation [46]. In the mitochondria, MTs exist in the mitochondrial intermembrane space (IMS) [43]. When Apo-MTs (thionein T), a metal-free MT, is introduced into the mitochondria, the suppressed cellular respiration by abnormal zinc influx can be restored [43]. However, free zinc or zinc coupled MTs suppresses mitochondrial function by inhibiting the mitochondrial electron transport system activity [43]. Thus, when the lysine residue of the β-domain of the N-terminal of MTs is carbamoylated, this inhibitory effect is prominent, and these residues have been reported to play an important role in the import of MTs through the outer space membrane of the mitochondria.

The function of MTs on the regulation of mitochondrial function has been demonstrated in various ways. For example, the viability of HeLa cells was improved by over-expressing MT-2A in the function of the mitochondrial electron transport system complex I inhibited by rotenone [47]. In MT knock out (KO) mice, hepatic ATP levels of the feeding cycle were significantly reduced [48]. MTs in the mitochondria regulates the expression of various genes involved in energy metabolism, such as *Gck* (glucokinase), *Elovl3* (very long chain fatty acid elongase), *Atp5a1* (ATP synthase), *Pdxk* (pyridoxal kinase), *Entpd5* (ectonucleoside triphosphate diphosphohydrolase 5) [49].

Unlike many reports of the role of MTs in mitochondria, little has been reported about the effects of MTs on lysosomes. However, there are some reports of MT3 in the regulation of lysosomal function. Lee et al. have reported on the role of MT3 in the regulation of lysosomal enzymes such as cathepsin L, cathepsin D, acid phosphatase, and neuramidase as well as lysosomal membrane proteins such as lysosome-associated membrane protein (Lamp) 1/2 [50]. In addition, MT3 null astrocytes indicated a slight increase in lysosomal pH (Fig. 2e). Alterations in lysosomal biogenesis in MT3 null astrocytes seem to result in part from abnormal localization and expression of vacuolar-type H^+^-ATPase subunit Voa1 (V-ATPase Voa1) (Fig. 2f), which is a highly conserved enzyme with the ability to pump proton on plasma membrane, as well as membranes of intracellular organelles. For this reason, when the cells exposed to H_2_O_2_, MT3 null astrocytes showed less lysosomal rupture, escaping from apoptotic cell death derived from released cathepsins (Fig. 2h). Moreover, astrocytes cultured form MT KO mice presented abnormal accumulation of filipin, a sterol-binding molecule, as well as lipofuscin, auto fluorescent lipid containing age pigment (Fig. 2j and k).

MT3 null astrocytes demonstrated abnormal distribution and morphology of endosomes and Golgi (Fig. 2b and c). However, the mechanism of these alterations needs to be clarified. Since this report, however, it was further discovered that MT3-induced lysosomal biogenesis can eventually control autophagy, a type of cell death [23, 50].

#### Regulation of autophagy and apoptosis by MT3

Autophagy plays an important role in the quality control of several intracellular biomolecules and organelles, and the function of lysosomes is in the center of the process of autophagy. An increase in the pH of the lysosome causes stagnation of autophagy, resulting in the accumulation of intracellular molecules [51]. In general, autophagy is necessary in normal cell life, and essentially, does not induce cell death. In fact, autophagy prevents the induction of apoptosis, and the activity of caspase stops the autophagy [52, 53]. However, in special cases, autophagy or autophagy-related proteins also help to cause apoptosis or necrosis. In increased abnormal stress, excessive cell breakdown is caused by excessive increase in autophagy, leading to autodigestive cell death. Thus, autophagy is closely related to apoptosis activated by mitochondria, and modulates various pathophysiological results. However, autophagy occurs primarily prior to apoptosis.

As described above, MT3 is involved in constant lysosomal pH control by moving zinc to lysosomes via a zinc transporter (ZIP8, SLC39A8), and in controlling the expression levels of the lysosomal membrane proteins LAMP1/2 by glycosylation, thus balancing the lysosomal biogenesis, making autophagy possible under various stress situations [50, 54]. When activated, this process contributes to inducing a smooth fusion of autophagosomes and lysosomes.

The mitochondria are known to play a central part in the intrinsic pathways of apoptosis. In many pathological conditions, apoptosis is over-stimulated, resulting in the destruction of excessive amounts of cells. In controlling of apoptosis, MTs is usually associated with controlling ROS; thus, overexpression of MTs can protects cells from the extreme cell death by scavenging and limiting of ROS [55]. This effect of MTs on apoptosis occurs through their interaction with cytochrome *c* or caspase-3, eventually contributing to the modification of cytochrome c or caspase-3 activity by certain metals in MTs like zinc [56]. Another report showed that MTs reduces drug-induced apoptosis more than p38 mitogen-activated protein kinase (MAPK) and cytochrome c-caspase 3 pathway inhibitors [57]. The reported functions of MTs on the mitochondria are primarily those other than MT3. However, recently, in pediatric acute myeloid leukemia, MT3 was shown to function as a putative tumor suppressor gene by inhibiting proliferation and inducing apoptosis via the upregulation of *forkhead box protein* 1 (*FOXO1*) [58]. Similarly, in cultured cortical astrocytes of the brain, zinc released from MT3 increased FOXO1/3 induction, thus activate p38 kinase and contributing to the activation of caspase9 and poly (ADP-ribose) polymerase (PARP), which leads to apoptosis [24]. In addition, H_2_O_2_ induces intracellular zinc increase from Zn-MT3 (Fig. 2I) and contributes to lysosomal rupture, causing the leaking of cathepsin D from lysosomes (Fig. 2g) and turning on the apoptosis of cortical astrocytes [50]. Further, cortical astrocytes in MT3 wild type mice show enlarged autophagosomes, implying defects in fusion of lysosomes and autophagosomes (Fig. 2i). Although it is not clear whether these effects are directly due to MT3 or from the zinc liberated from MT3, these reports show that Zn-MT3 may control intracellular homeostasis by functioning as an important regulatory molecule in apoptotic and autophagic death in cortical astrocytes.

### MT3 and diseases

**Fig. 3:**
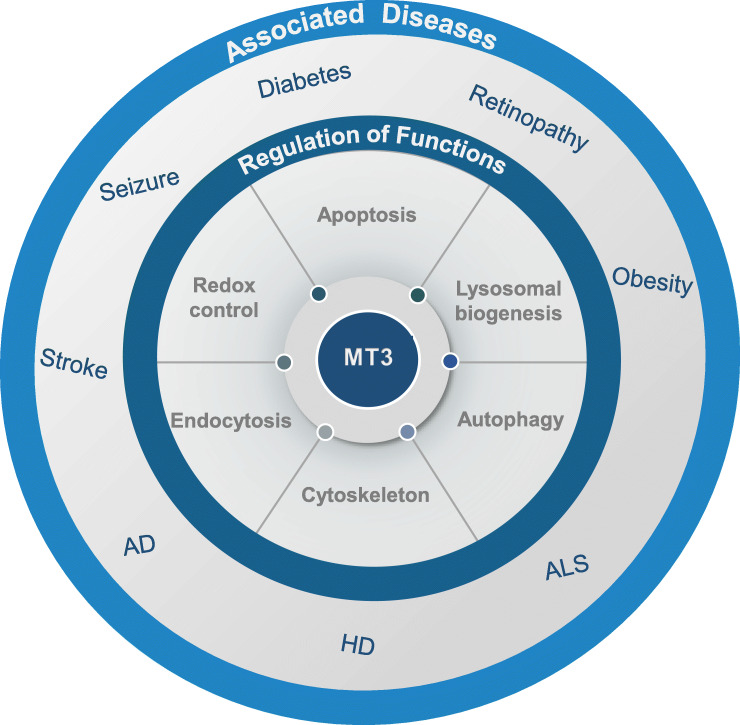
Summary diagrams depicting MT3 effects on diseases. MT3 contributes to the normal progression of redox reaction, lysosomal biogenesis, autophagy, apoptosis, endocytosis, and cytoskeleton, which are all beneficial for cell survival. However, altered expression of MT3 in cortical astrocytes of null mice leads to various pathological conditions, such as Acute and chronic neuronal diseases, cancer, retinopathy, and diabetes. AD, Alzheimer disease; HD, Huntington disease; ALS, amyotrophic lateral sclerosis; AMD, age-related macular degeneration

As described earlier, MT3 has a variety of functions in various cellular compartments, such as apoptosis based on ROS regulation, autophagy via lysosomal biogenesis regulation, and endocytosis control through the regulation of cytoskeleton dynamics. Therefore, Fig. 3 examines the diverse diseases that may occur when MT3 is malfunctioned or dysregulated.

#### Brain injury and MT3

The primary function of MTs is to scavenge ROS produced under various stresses in cells. MT3, a CNS dominant form of MTs, has been reported as one of the causative proteins in neuronal death following ischemic brain injury [59, 60]. In these cases, released zinc from Zn-MT3 in the injured brain may play a key role in the progression of cellular death, and application of zinc chelator, calcium ethylenediamine tetraacetic acid (CaEDTA) or N, N, N’N′-tetrakis-(2-puridylmethyl) ethylenediamine (TPEN), prevented intracellular zinc release/accumulation in neurons [59].

As an inhibitor of neuronal growth (Fig. 2a), MT3 is also expected to play an important role in various neurodegenerative disease. Indeed, MT3 shows abnormal expression in each lesion in patients with Alzheimer’s disease (AD) as confirmed by immunohistochemistry, Northern blotting, and reverse transcription polymerase chain reaction (RT-PCR) [7, 61]. MT3 is predominantly present in neurons particularly with high concentrations of zinc. However, it was reported that MT3 expression is significantly changed in the astrocytes of patients with AD or PD [62, 63]. The exact mechanism of this alteration has not been clarified in detail. Recently, our group found that MT3 KO astrocytes presented abnormal localization and expression of presenilin and mHttQ74 compared to WT cells (Fig. 2l and m).

Neurodegeneration depends on the region affected and the lamina of the cerebral cortex, as well as the clinical stages of the disease. Neurofibrillary tangles (NFTs) formation and the resulting neurodegeneration starts in the hippocampus, particularly, in pyramidal neurons in CA1 and layer II of the entorhinal cortex [62, 63]. However, no cognitive impairment or nerve loss is observed at this time. Afterwards, NFTs gradually spread from the limbic system to the neocortex. Specifically, MT3 expression is partially reduced in astrocytes of the laminar rather than in the entire brain, especially in the superficial layer of AD gray matter [62, 63]. Expression level of MT3 protein and mRNA are also significantly reduced in astrocytes in the vicinities of senile plaques in AD, Down’s syndrome, and Kuru plaques in Creutzfeldt-Jakob disease (CJD). Thus, MT3 reduction is associated with neuronal loss or disease duration, with its mRNA or protein expression in lesions reduced in proportion corresponding to the clinical stage of AD. Therefore, the amount of MT3 expression in the brain of patients with AD may be a clinical indicator of the disease.

Similar to AD, in patients with ALS, the degree of immunostaining for MT3 in astrocytes is closely related to the number of motor neurons in the ventral horn [7]. One of causes of familial ALS is mutations in copper-zinc superoxide dismutase (SOD1) gene [64]. During the initial progress in ALS, MT3 expression increases and MT-1/2 and MT3 knock-out mice showed the reduced survival time and accelerated onset and progression of ALS [65]. Low expression of MT3 has been also reported in the brain of Parkinson’s disease (PD) [66]. The important factors for PD are the formation of ROS and dopamine quinones, contributing to pathogenesis of PD and characterized by dopamine depletion in the basal ganglia and by dopaminergic cell death in the substantia nigra pars compacta [67]. Studies showed that dopamine increases ROS in the glial cell line, thereby increasing MT3 mRNA expression [68]. 6-hydroxydopamine (6-OHDA) also increase striatal MT3 expression, considered an initial response to compensate for oxidative stress [66]. However, when levodopa was administered to the 6-OHDA-lesioned side in PD, there was no change in MT3 expression. Therefore, in the parkinsonian brain, MT3 expression with free radical scavenging potency was rather reduced, which contributes to accelerated PD progression. Similar result was shown in MT-1/2 KO mice demonstrating possible neuroprotective effects of MTs in the PD. MT3 expression abnormalities have been reported in various neuropathic diseases, but little has been reported about the detailed molecular mechanisms of action, excluding its ROS inhibitory activity. Therefore, it is necessary to study the mechanism of action of MT3 on diverse neurodegenerative diseases.

#### Diabetes and MT3

Diabetes is gradually increasing in the world due to changes in living environment and high fat and high-calorie Western diet. Diabetes breaks down the proper circulation and balance of glucose, causing the alteration of body’s metabolic system, leading to multiple complications such as retinopathy, nephropathy, and cancer. Therefore, vision loss or kidney failure may result [69]. Diabetes also causes diseases affecting lifespan, such as cerebral infarction and coronary artery disease [70].

β-islet cells of the pancreas play the most important role in controlling blood sugar and have an exceptionally reduced activity of antioxidant enzymes that produce ROS, which rapidly increases as a metabolic product during hyperactive intracellular metabolism and may lead to cell death [71]. In fact, MTs protect β-islet cells from oxidative stressors such as nitric oxide, peroxynitrite, hydrogen peroxide, superoxide, and streptozotocin (STZ). As a result, β-islet cells in animal models of obesity and type 2 diabetes induced by a high fat diet increases blood sugar control by smooth insulin secretion, thereby reducing disease severity. In particular, in humans, polymorphisms of *MT1A* and *MT2A*, the genes encoding MTs, showed a greater risk of promoting the progression of type 2 diabetes and complications of diabetes [72]. In addition, the amount of pancreatic and duodenal homeobox 1 (PDX1) expression, which is a fatal regulator of pancreatic β cell differentiation, survival, and insulin synthesis, was also abnormally changed in transgenic mice overexpressing MT specifically in β cells, and the expression of genes regulated by PDX1, including *Ins1* (insulin-1 precursor), *Gck*, and *Glut2* (glucose transporter 2), is also changed [71].

Besides the CNS, MT3 is also expressed in non-CNS locations such as pancreas, prostate, testis, and tongue [73]. The pancreas is composed of two types cells, which are endocrine islets and exocrine acinar cells, and pancreatic islets are derived from neuroectoderm and share similarities in development with the brain; the islets express γ-aminobutyric acid (GABA), neuropeptide-Y, and brain-derived neurotrophic factor (BDNF), as in the brain [74–76]. Specifically, MT3 mRNA is highly expressed in pancreatic islets [73], in high association with Zn dyshomeostasis. According to a previous report by Byun et al. [77], MT3 also regulates phosphodiesterase 3a (PDE3a), an enzyme that regulates levels of cAMP and cGMP in diabetes. MT3-null mice were less sensitive to STZ- and sodium nitroprusside (SNP)-induced toxicity due to reduced Zn release associated with oxidative toxicity and decreased PDE3a expression or activity [77]. In addition, cardiomyopathy accompanied by diabetes could also be reduced by overexpression of MTs [78].

#### Retinopathy and MT3

The eye is continuously exposed to various kinds of light sources that may easily cause damage by oxidative stress. All ocular structures consist of the posterior pole, the neurosensory retina, and the retinal pigment epithelium (RPE). With age, RPE can be suddenly damaged in response to oxidative stressors, resulting in age-related eye diseases such as diabetic retinopathy, retinitis pigmentosa, and age-related macular degeneration (AMD) [79, 80].

It has been proven that MTs in the human eye are highly expressed throughout the ocular tissues, specifically in the cornea, lens, and RPE [81]. These areas are the ocular natural barriers protecting against environmental insults. MTs contribute to the cellular ROS defense system by buffering intracellular labile zinc, thus preventing the eye from the AMD progress [82]. Similarly, zinc rise due to tamoxifen treatment causes induction of autophagy and this leads to cell death in cultured retinal epithelial and photoreceptor cells [83]. However, in these cases, it is still not clear whether the ROS induced by zinc originates from MTs. High content of sulfhydryl clusters of MTs are the main site for ROS scavenging and swapping. Reflecting this, MT3 deficiency exacerbates light-induced retinal degeneration [84]. In this case, the protective effect of MT3 was higher than that by MT-1/2.

#### Cancer and MT3

Cancer is often caused by problems with the normal metabolic processes of cells and has the high levels of metabolic activity. Metabolic abnormalities caused by various stressors from endogenous or exogenous origins increase ROS, accumulating a bulk of free radicals in the cells. This causes mutation of DNA and various large molecules essential for survival, sometimes leading to irreversible cell proliferation following inexorable abnormal activity of cell cycle [85]. Therefore, removing the abnormal ROS generated in the early stage of stress is the first defense mechanism to prevent carcinogenesis.

As described earlier, MT3 is excellent in its ability to swap various toxic metals or radicals, due to its biochemical structure. Therefore, cells can be more quickly avoid stress when the expression level of MT3 is increased in an initial oxidative stress situation. However, the responses of many tumor cells and tissue under oxidative stress conditions are still controversial. This is because results vary depending on the stages of the tumor progress. When the ROS produced by excessive metabolism of tumor cells increases, such that it cannot be removed by MT3 or antioxidants, it activates the immune response of surrounding cells to induce inflammation, thus either abnormally increases cell activity or leading to death. In addition, in early cancer, in general, activated autophagy functions as a tumor suppressor, but once cells become malignant and advanced, it seems to contribute to tumor survival [86]. Therefore, autophagy is often induced when cancer is treated with radiation therapy, various hormone, or chemotherapy, and may have side effects that eventually contribute to the survival of cancer cells. Many studies report that MT3 gene knock out is effective in preventing recurrence of tumor due to side effects of these therapies. For example, MT3 gene knock out showed an effect on post-radiation induced survival of glioma cells [87]. In MCF-7 cells, a breast cancer cell line, the C- and N-terminal of MT3 contributed to the growth inhibition of cancer cells [88]. Furthermore, MTs expression level or polymorphisms are used to diagnose risks of various cancers such as gastric cancer, colorectal cancer, breast cancer, and hematological malignancies, or to predict cancer progression [89].

## Conclusion

MT3 exists in various organs but with dominant abundance in the brain. The most important function of MT3 known so far is its antioxidant action via the oxidation-reduction reaction under the oxidative stress conditions. This function is due to the molecular structural properties of MT3, which prevent the damages of cellular organelles and biomolecules, such as carbohydrates, proteins, and lipid derivatives. Especially, the function of MT3 in oxido-reduction reaction is important in suppressing the early stages of various diseases such as stroke, cardiomyopathy, diabetic retinopathy, and cancer. Therefore, studies on the MT3 polymorphisms in various disease are the subject of active research.

In addition to oxidative stress control, which is a feature common to other MT isoforms, MT3 regulates autophagy. The lysosome is a very important organelle in autophagy. Although many reports have described internal and external factors that control autophagy, final destination of the autophagy is lysosome, a place where various cargo contents are decomposed. A variety of attempts have been made to induce cancer cell death by reducing the flow of autophagy, using many therapeutic agents that modulate lysosomal function. In particular, the absence or reduction of MT3 weakens many functions of lysosomes, such as decrease enzymatic activity due to the change in lysosomal pH, thus preventing fusion with autophagosomes, which is the final step of autophagy, and this contributes to the destruction of the normal recycling of cellular contents. MT3 also participates in changes in cell metabolism and cellular signaling systems, such as cytoskeleton modification and polymerization, vesicular trafficking of signaling substances via endocytosis or exocytosis, arrangement of endosomes, and formation of Golgi structure, which may in turn lead to maintenance of normal cellular activities or control of multiple diseases. Therefore, in-depth study on the development of customized treatments targeting MT3 in various diseases are needed in the future.

## Data Availability

Not applicable.

## Abbreviations

Aβ: Amyloid beta
AD: Alzheimer’s disease
ALS: Amyotrophic lateral sclerosis
AMD: Age-related macular degeneration
Apo-MT: Thionein T
Atp5a1: ATP synthase
BDNF: Brain-derived neurotrophic factor
CaEDTA: Calcium ethylenediamine tetraacetic acid
CJD: Creutzfeldt-Jakob disease
CK: Creatine kinase
DRP-2: Dihydropyrimidinase-like protein 2
eEF1α: Elongation factor 1α
EGF: Epidermal growth factor
Elovl3: Very long chain fatty acid elongase
Entpd5: Ectonucleoside triphosphate diphosphohydrolase 5
F-actin: Fibrillary actin
FOXO1: Forkhead box protein 1
GABA: γ-aminobutyric acid
Gck: Glucokinase
GIF: Growth inhibitory factor
Glut2: Glucose transporter 2
HD: Huntington’s disease
HSP84: Heat-shock protein 84
6-OHDA: 6-Hydroxydopamine
Ins1: Insulin-1 precursor 1
KO: Knock out
Lamp1: Lysosome-associated membrane protein 1
MAPK: Mitogen-activated protein kinase
MT: Metallothionein.
NFTs: Neurofibrillary tangles.
NO: Nitric oxide.
PD: Parkinson’s disease.
PARP: Poly (ADP-ribose) polymerase.
PDE: Phosphodiesterase.
PDX1: Pancreatic and duodenal homeobox 1.
Pdxk: Pyridoxal kinase.
ROS: Reactive oxygen species.
RPE: Retinal pigment epithelium.
RT-PCR: Reverse transcription polymerase chain reaction.
SOD: Superoxide dismutase.
SNP: Sodium nitroprusside.
STZ: Streptozotocin.
TPEN: N,N,N’N′-tetrakis-(2-puridylmethyl) ethylene diamine.
UTR: Untranslated region.
V-ATPase-Voa1: Vacuolar-type H^+^ ATPase subunit Voa1.
ZIP: Zinc transporter
